# Repetitive transcranial magnetic stimulation ameliorates cognitive deficits in mice with radiation-induced brain injury by attenuating microglial pyroptosis and promoting neurogenesis via BDNF pathway

**DOI:** 10.1186/s12964-024-01591-0

**Published:** 2024-04-03

**Authors:** Tongzhou Qin, Ling Guo, Xing Wang, Guiqiang Zhou, Liyuan Liu, Zhaowen Zhang, Guirong Ding

**Affiliations:** 1 Department of radiation protection medicine, School of Preventive Medicine, Fourth Military Medical University, Xi’an, 710032 China; 2 Ministry of Education Key Lab of Hazard Assessment and Control in Special Operational Environment, Xi’an, China; 3 Department of occupational & environmental health, School of Public Health, Weifang Medical University, Weifang, 261021 China

**Keywords:** rTMS, RIBI, BDNF, Neuroinflammation, Neurogenesis

## Abstract

**Background:**

Radiation-induced brain injury (RIBI) is a common and severe complication during radiotherapy for head and neck tumor. Repetitive transcranial magnetic stimulation (rTMS) is a novel and non-invasive method of brain stimulation, which has been applied in various neurological diseases. rTMS has been proved to be effective for treatment of RIBI, while its mechanisms have not been well understood.

**Methods:**

RIBI mouse model was established by cranial irradiation, K252a was daily injected intraperitoneally to block BDNF pathway. Immunofluorescence staining, immunohistochemistry and western blotting were performed to examine the microglial pyroptosis and hippocampal neurogenesis. Behavioral tests were used to assess the cognitive function and emotionality of mice. Golgi staining was applied to observe the structure of dendritic spine in hippocampus.

**Results:**

rTMS significantly promoted hippocampal neurogenesis and mitigated neuroinflammation, with ameliorating pyroptosis in microglia, as well as downregulation of the protein expression level of NLRP3 inflammasome and key pyroptosis factor Gasdermin D (GSDMD). BDNF signaling pathway might be involved in it. After blocking BDNF pathway by K252a, a specific BDNF pathway inhibitor, the neuroprotective effect of rTMS was markedly reversed. Evaluated by behavioral tests, the cognitive dysfunction and anxiety-like behavior were found aggravated with the comparison of mice in rTMS intervention group. Moreover, the level of hippocampal neurogenesis was found to be attenuated, the pyroptosis of microglia as well as the levels of GSDMD, NLRP3 inflammasome and IL-1β were upregulated.

**Conclusion:**

Our study indicated that rTMS notably ameliorated RIBI-induced cognitive disorders, by mitigating pyroptosis in microglia and promoting hippocampal neurogenesis via mediating BDNF pathway.

**Supplementary Information:**

The online version contains supplementary material available at 10.1186/s12964-024-01591-0.

## Introduction

Radiation-induced brain injury (RIBI) is a common and serious complication of cranial radiotherapy, it mainly manifested as cognitive decline, emotional alteration and locomotive dysfunction, which greatly affected patients’ quality of life. The incidence of RIBI is increasing, while its underlying mechanisms are complicated and not fully clarified. It has been reported that RIBI can inhibit the proliferation of neural stem cells (NSCs) in hippocampus and the ability of differentiating into mature neurons, radiation can also activate microglia in brain and induce neuroinflammation [1, 2]. Besides, cranial irradiation leads to destruction of blood-brain barrier (BBB), DNA damage and cell death through promoting the generation of free radicals and reactive oxygen species (ROS) in the brain [3, 4] Due to the poor prognosis and unclear mechanism with no effective measures for the treatment of RIBI, it is pivotal to seek for a novel method to deal with this complication.

Repetitive transcranial magnetic stimulation (rTMS) is a novel non-invasive brain stimulation method with few side effects. Recently, rTMS has gained wide attention in the treatment of central nervous system (CNS) diseases, such as Parkinson’s disease, Alzheimer’s disease, stroke and depression, et al. [5–7]. It had been reported that rTMS could improve cognitive function by regulating synaptic plasticity [8], promoting hippocampal neurogenesis [9] and alleviating neuroinflammation in the brain [10]. A rTMS clinical guideline conducted by Lefaucheur et al. revealed that neuropathic pain, depression and subacute stroke were classified as “Grade A” recommendation with definite efficacy [11]. However, the potential mechanism of rTMS on CNS diseases treatment still remain unclear, besides, the clinical therapeutic effects of rTMS on RIBI are rarely reported.

Brain-derived neurotrophic factor (BDNF), one of the most important neurotrophic proteins in CNS, exerts neuroprotective effect on the repairment of inflammation and ischemic stress, and is proved to decrease the level of apoptosis [12]. Guo et al. found that rTMS could mitigate cognitive dysfunction through promoting neurogenesis and inhibiting neuronal apoptosis in hippocampus, which may be related to the activation of BDNF-TrkB pathway [13]. In addition, Luo et al. recently revealed that high-frequency rTMS significantly improved neural function recovery and promoted neurogenesis in ischemic stroke rat model [14]. In our previous study, we firstly reported that rTMS had preventive and therapeutic effects on RIBI mice, which were manifested by alleviation of cognitive dysfunction of RIBI mice after rTMS intervention [15], and BDNF may play a critical role in it. However, the specific role of BDNF in the prevention and treatment of RIBI has not been fully elucidated.

In the present study, we explored the potential mechanisms of rTMS by verifying the hippocampal neurogenesis and neuroinflammation on RIBI mice, and detected the role of BDNF in it. Based on these results, we further investigated whether pretreatment with K252a, an agonist of BDNF pathway, could reverse the improvement of cognitive function in RIBI mice after rTMS treatment. In addition, we assessed the possible role of BDNF on cognitive function, hippocampal neurogenesis and neuroinflammation in RIBI mice model.

## Materials and methods

### Animals

Healthy male C57BL/6 mice (6–8 weeks, Laboratory Animal Center of Fourth Military Medical University) were kept in controlled condition with 12-h light and 12-h dark cycle (lights on from 8:00 A.M. to 8:00 P.M.), temperature 23 ± 2℃ and humidity 50 ± 2%, and had free access to food and water. Before the experiment, all the mice were allowed to adapt to the conditions for 1 week, then the mice were randomly assigned to Sham irradiation group (Sham), cranial irradiation group (Radiation) and rTMS intervention group (rTMS), with 12 mice in each group. All the animal procedures in this study were conducted in accordance with the ethical guidelines of Animal Welfare Committee of Fourth Military Medical University (IACUC-20210105).

### Cranial irradiation and rTMS intervention protocol

The protocols of cranial irradiation and rTMS intervention have previously been reported [16]. Briefly, the mice were kept in a fixator to remain stable and conscious, and received cranial X-ray irradiation 5 Gy/d for consecutively 4 d at a dose rate of 2.33 Gy/min. The other parts of body were shielded by lead plate. The mice in the Sham group were subjected to the same protocol as that of radiation group, while the irradiator was switched off. After cranial irradiation, rTMS intervention was performed immediately. High-frequency (10 Hz) rTMS was applied with a round prototype coil, and the mice were awake and handled gently. The parameters are composed of two sessions with 120 s interval, and each session consisted of 500 pulses.

### Chemicals and treatment

In order to examine the function of BDNF pathway in the therapeutic effects of rTMS, K252a intervention group (Radiation + rTMS + K252a) was used in subsequent experiment, which was termed as K252a group. K252a (Meilunbio, China), a specific receptor antagonist of TrkB, was dissolved at a dose of 25 μg/kg in saline containing 1% DMSO and intraperitoneally injected daily after cranial irradiation. K252a was injected to the mice in K252a group, and mice in other groups were administrated 0.9% saline via intraperitoneally injection. Besides, 5-bromo-2’-deoxyuridine (BrdU, Sigma-Aldrich, USA) was used to label S-phase proliferated cells in hippocampus. Briefly, BrdU (50 mg/kg body weight) was daily intraperitoneally injected to 3 mice in each group for consecutively 3 days before sample collection.

### Behavioral tests

#### Morris water maze (MWM)

The long-term spatial memory ability was assessed by MWM as previously described [17] with some proper modifications. Briefly, the maze was divided into four quadrants and the platform was placed in one of the quadrants beneath the water 1–2 cm during the training phase. Besides, milk was used to make the water invisible to the mice. During the positioning navigation experiment, each mouse was gently released into the water and allowed to find the platform within 60 s, if the mouse failed to find the platform, it was guided to the platform and was allowed to stay for 15 s. After the navigation phase, the platform was removed, each mouse was released into the water and allowed to swim freely from the same starting position for 60 s. The escape latency and the time spent in target quadrant were recorded and analyzed using an animal tracking system (Ethovision XT 15.0, Holland).

#### Open field test (OFT)

OFT was performed to evaluate the anxiety-like behavior of mice. During the test, each mouse was gently placed in the open field apparatus (50 cm × 50 cm × 40 cm) and allowed to explore freely for 5 min. After each session, 75% alcohol solution was used to wipe the apparatus and clean the feces and urine to prevent the odor traces. The animal activity in the open field was recorded by animal tracking system (Ethovision XT 15.0, Holland). The accumulative locomotion distance, total time spent in the central areas were calculated and analyzed.

#### Elevated plus-maze test (EPM)

The apparatus of EPM was composed of two open arms and two closed arms, which was usually used to assess the anxiety-like and exploring behavior of mice. Each mouse was gently placed in the center of the apparatus, facing to the open arms, and was allowed to explore the maze freely for 5 min. The activity of each mouse was recorded with animal tracking system. After each session, all the feces and urine were completely cleaned and maze was wiped with 75% alcohol solution. The total time spent in the open arms and number of open arms entries were calculated and analyzed.

### Immunofluorescence (IF) staining

After routine deparaffinization and rehydration, the paraffin brain sections were processed by antigen retrieval and treated with 10% normal goat serum for 1 h at room temperature to block nonspecific binding sites. Then the sections were incubated with primary antibodies overnight at 4℃. The details of primary antibodies are shown in Table S1. Next, the brain sections were rinsed with PBS and incubated with Alexa Fluor 488 or Alexa Fluor 594 secondary antibodies (1:200, Zhuangzhi Bio, China) for 1 h at room temperature. Nuclei were stained with DAPI for 5 min at room temperature. The experiment was repeated twice and the images were captured by confocal microscope (Nikon, Japan).

### Immunohistochemistry (IHC) staining

Paraffin brain sections were processed by antigen retrieval using citrate buffer (pH = 6.0) at high temperature for 10 min, and washed 3 times with PBS at room temperature. The sections were blocked with normal goat serum for 30 min at 37℃, and incubated with primary antibody Iba-1 (Rabbit mAb, 1:300, Proteintech, USA) overnight at 4℃. Next, the brain sections were incubated with corresponding secondary antibody (Chromogenic reagent HRP, mouse/rabbit, MXB Biotech) for 30 min at room temperature. The DAB working solution was added to react for 1–3 min, and observed under the optical microscope. Hematoxylin solution was added to the sections for 5 min, and reacted with 0.5% hydrochloric alcohol solution for 3 s before ammonia solution for 1 min. Finally, all the sections were successively rinsed into 75%, 95% and 100% alcohol. It was repeated 3 times, and the images were collected under the optical microscope (Leica, Germany).

### Western blotting analysis

We performed 10–12% sodium dodecyl sulfate-polyacrylamide gel electrophoresis (SDS-PAGE) as conventional procedure. Briefly, the protein samples were separated by SDS-PAGE and transferred onto PVDF membranes (Millipore, USA). Then the membranes were blocked with 5% nonfat milk for 2 h at room temperature and incubated with the primary antibodies overnight at 4℃. The details of antibodies are listed in Table S1. After washing 3 times in TBST, the membranes were incubated with corresponding secondary antibodies for 1 h at room temperature. It was repeated 3 times and the bands were detected and visualized by ECL reagent (Beyotime, China). The immunoblots were analyzed by Image J software.

### Golgi staining

The Golgi staining was conducted strictly according to the manual of Rapid Golgi Staining Kit (FD Neurotech, USA). Briefly, the freezing brain tissues were quickly coronally sliced with the thickness of 150 μm at -20℃, using a freezing microtome (Leica, Germany). Then the brain sections were stained based on the protocols, the dendritic spine in the middle of tertiary dendrites of pyramidal neurons in DG region of hippocampus was captured by the optical microscope (Olympus, Japan). It was repeated twice, and a total of 6 non-overlapping different fields were randomly captured from 2 independent animals.

### Statistical analysis

All the data were presented as mean ± standard error of mean (S.E.M). The data were analyzed with SPSS 22.0, and GraphPad Prism 9.0 (GraphPad Software, Inc., La Jolla, CA, USA) was used for graphing. Normality of data was examined first by Shapiro-Wilk test, and the differences among each group were examined by one-way analysis of variance (ANOVA) followed by Tukey test for multiple comparison. Escape latency was compared by two-way ANOVA. For non-normal distribution data, non-parametric analysis was used. The difference was considered statistically significant when the *P* value was less than 0.05.

## Results

### rTMS promoted hippocampal neurogenesis in RIBI mice

**Fig. 1:**
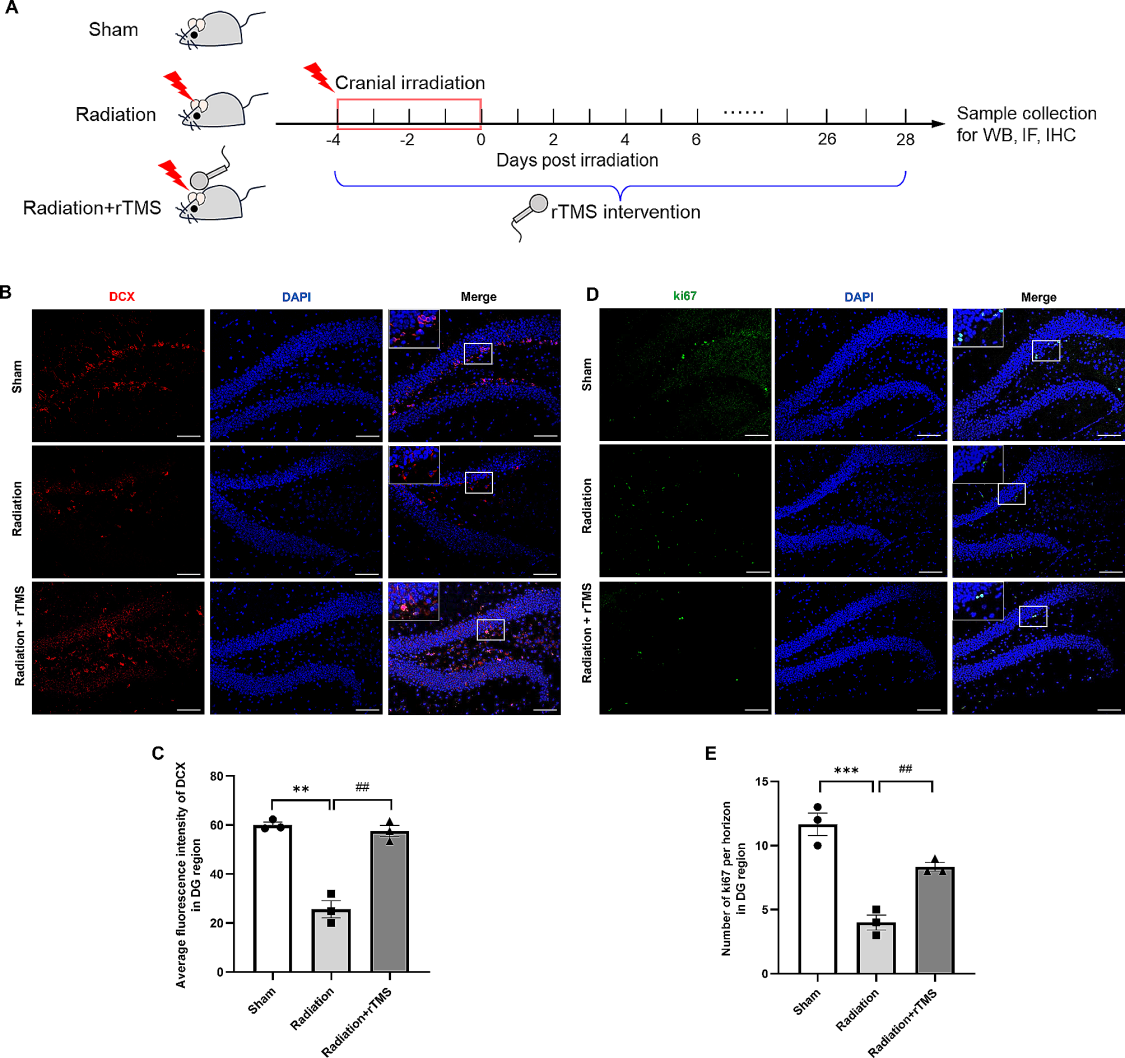
rTMS ameoliated hippocampal neurogenesis impairment induced by cranial irradiation. (**A**) Schematic diagram of the experimental design, including animal grouping, radiation and rTMS intervention protocol. (**B, D**) Representative immunofluorescence staining images of DCX and ki67 in hippocampus. Scale bar = 100 μm. (**C**) The average fluorescence intesity of DCX in DG region of hippocampus. (**E**) The number of ki67 in DG region. *n* = 3 for each group. Data are presented as mean ± S.E.M, ^**^P < 0.01, ^***^P < 0.001 vs. Sham group; ^##^*P* < 0.01 vs. Radiation group

We established the RIBI model as previously described [15], rTMS intervention was applied during and after radiation process, the details of radiation and rTMS intervention procedure were shown in Fig. 1A. It was found that the DCX fluorescence density of radiation group was significantly lower than that of Sham group, and the difference was statistically different. rTMS intervention could significantly increase DCX density in region of hippocampus (Fig. 1B, C). The ki67 labeled cells represented the proliferation of cells in DG region of hippocampus. The result of IF staining also showed that the number of ki67 in DG significantly increased compared with that of radiation group (Fig. 1D, E). The results above suggested that rTMS could promote hippocampal neurogenesis in RIBI mice.

### rTMS attenuated neuroinflammation in RIBI mice via reducing NLRP3 inflammasome-induced microglial pyroptosis

**Fig. 2:**
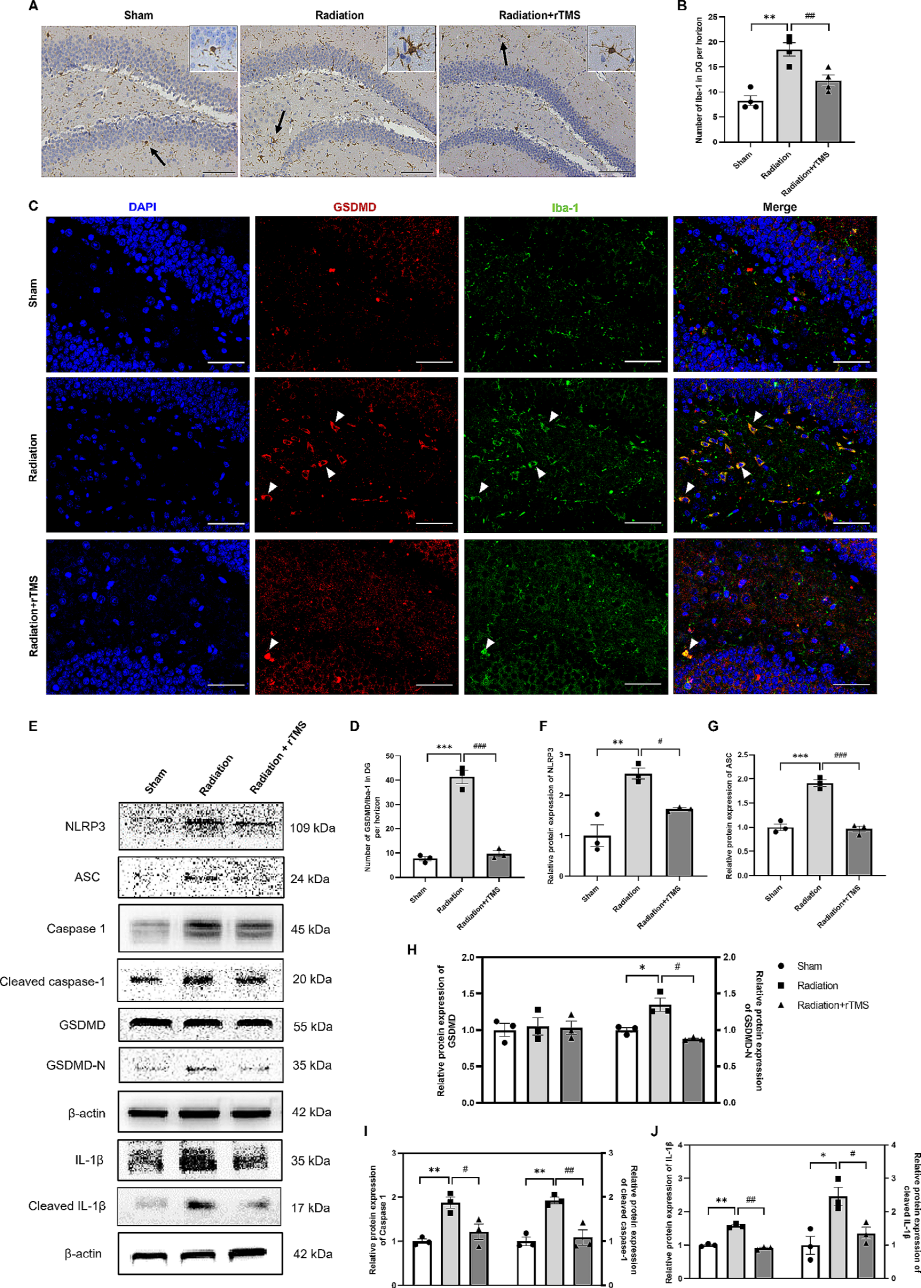
rTMS attenuated microglia pyroptosis induced by NLRP3 inflammasome. (**A**) Immunohistochemical staining of Iba-1 for indication of microglia in hippocampus. Scale bar = 100 μm, the enlarged image indicated the morphology of microglia. (**B**) The number of Iba-1^+^ in DG region. Data are presented as mean ± S.E.M. (**C**) Immunofluorescence staining of GSDMD and Iba-1, the arrows indicate co-localization of GSDMD with Iba-1. Scale bar = 50 μm. (**D**) The quantitative analysis of number of GSDMD^+^/Iba-1^+^ in DG region. (**E-J**) Western blotting representative bands and quantitative analysis. Protein expression level was normalized to β-actin. The data are presented as mean ± S.E.M, *n* = 3 for each group. ^*^P < 0.05; ^**^P < 0.01; ^***^P < 0.001 vs. Sham group, ^#^*P* < 0.05; ^###^*P* < 0.001 vs. Radiation group

To evaluate the level of neuroinflammation after rTMS intervention in RIBI mice, we observed the number and morphology of microglia in hippocampus, as the activation of microglia was pivotal in inflammatory response through pyroptosis [18, 19]. It was found that cranial irradiation induced microglial activation in DG region of hippocampus, compared with Sham group, the number of microglia significantly increased. After rTMS intervention, the microglia showed smaller size and thinner branches, and the number of which significantly reduced (Fig. 2A-B). We then detected the expression levels of Iba-1 and GSDMD by double-labeled immunofluorescence staining (Fig. 2C-D). We found that the number of GSDMD^+^/Iba-1^+^ cells significantly elevated after cranial irradiation, compared with that of Sham group. However, there was no obvious double labeled cells between GSDMD and NeuN or GFAP, which indicated that elevated GSDMD was mainly concentrated in the cytoplasm of microglia, not neurons or astrocytes (Figure S1). Besides, rTMS intervention could significantly decrease the number of GSDMD^+^/Iba-1^+^ cells, which suggested that rTMS could mitigate microglial pyroptosis in RIBI mice.

It has been reported that NLRP3 inflammasome activates caspase-1, and induces transmembrane pores, which contributes to the release of inflammatory cytokines, excessive NLRP3 inflammasome activity leads to cell pyroptosis [20–22]. It was found that cranial irradiation significantly upregulated the level of NLRP3 in hippocampus of mice, compared with that of in Sham group. rTMS could inhibit the expression of NLRP3, and the level of NLRP3 significantly decreased in rTMS intervention group, compared with radiation group (Fig. 2E-F). We also found that the levels of ASC and caspase-1 obviously increased in hippocampus of RIBI mice, which could be reversed by rTMS intervention (Fig. 2E, G, I). There was no obvious difference between radiation and Sham group in the expression level of GSDMD, while the expression of GSDMD-N significantly increased in radiation group, and rTMS intervention could reverse the upregulation of GSDMD-N (Fig. 2H). The pyroptosis process promotes the release of several proinflammatory cytokines, such as IL-1β. We found the protein expression level of IL-1β in hippocampus significantly increased after cranial irradiation and decreased after rTMS intervention (Fig. 2J). These results suggested that rTMS intervention could mitigate microglia-pyroptosis induced by NLRP3 inflammasome activation.

### rTMS activated BDNF signaling pathway after cranial irradiation

**Fig. 3:**
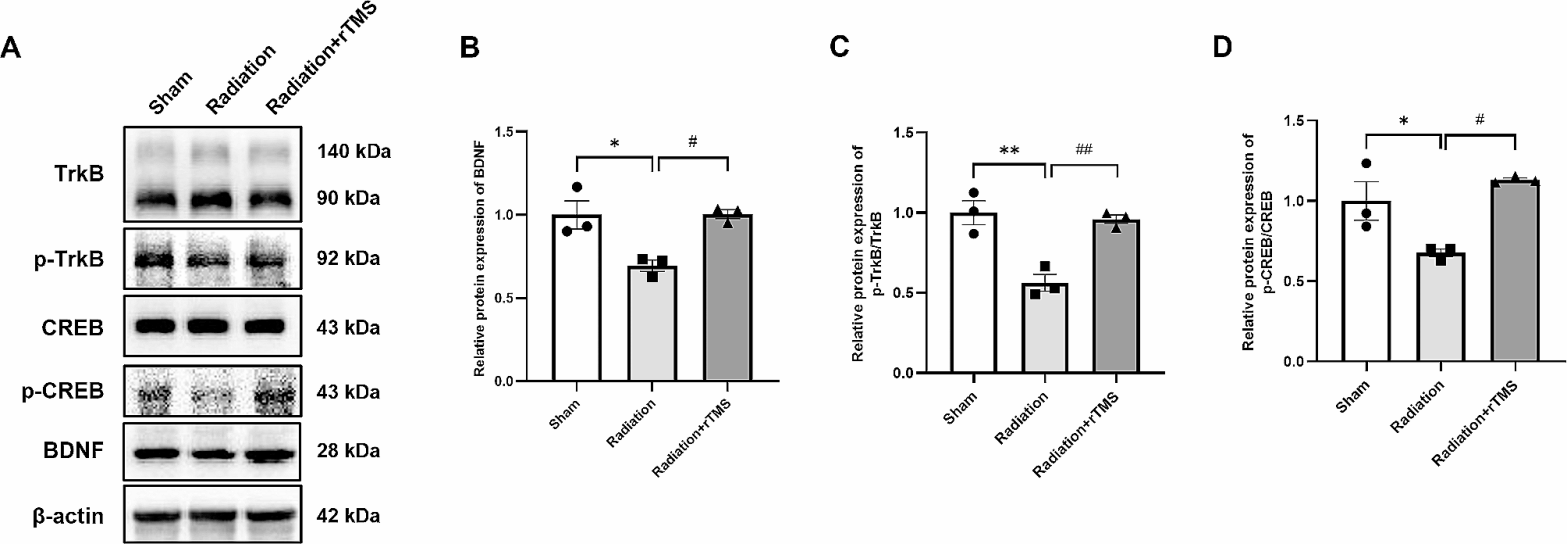
rTMS activated BDNF signaling pathway in hippocampus after RIBI. (**A**) Representative images of western blot bands. (**B-D**) The relative protein expression level of BDNF, p-TrkB/TrkB and p-CREB/CREB. Data are presented as mean ± S.E.M, *n* = 3 for each group. ^*^P < 0.05, ^**^P < 0.01 vs. Sham group; ^#^*P* < 0.05, ^##^*P* < 0.01 vs. Radiation group

It has been reported that BDNF is involved in neurogenesis and long-term potentiation (LTP) and it regulates learning and memory ability [23–25]. We previously found that large fractionated and conventional fractionated doses of X-ray irradiation could downregulate BDNF expression in hippocampus, and rTMS intervention increased the level of BDNF expression in RIBI mice. In this study, we found that total TrkB and CREB levels were consistent among each group, but the expression levels of BDNF, p-TrkB/TrkB and p-CREB/CREB ratio significantly decreased, compared with that of Sham group (Fig. 3A). rTMS could increase the expression level of these proteins, and the differences were statistically significant (Fig. 3B-D). These results indicated that BDNF pathway may be involved in neuroprotective functions of rTMS in RIBI mice.

### rTMS mediated cognitive function and anxiety-like behavior in RIBI mice through BDNF pathway

**Fig. 4:**
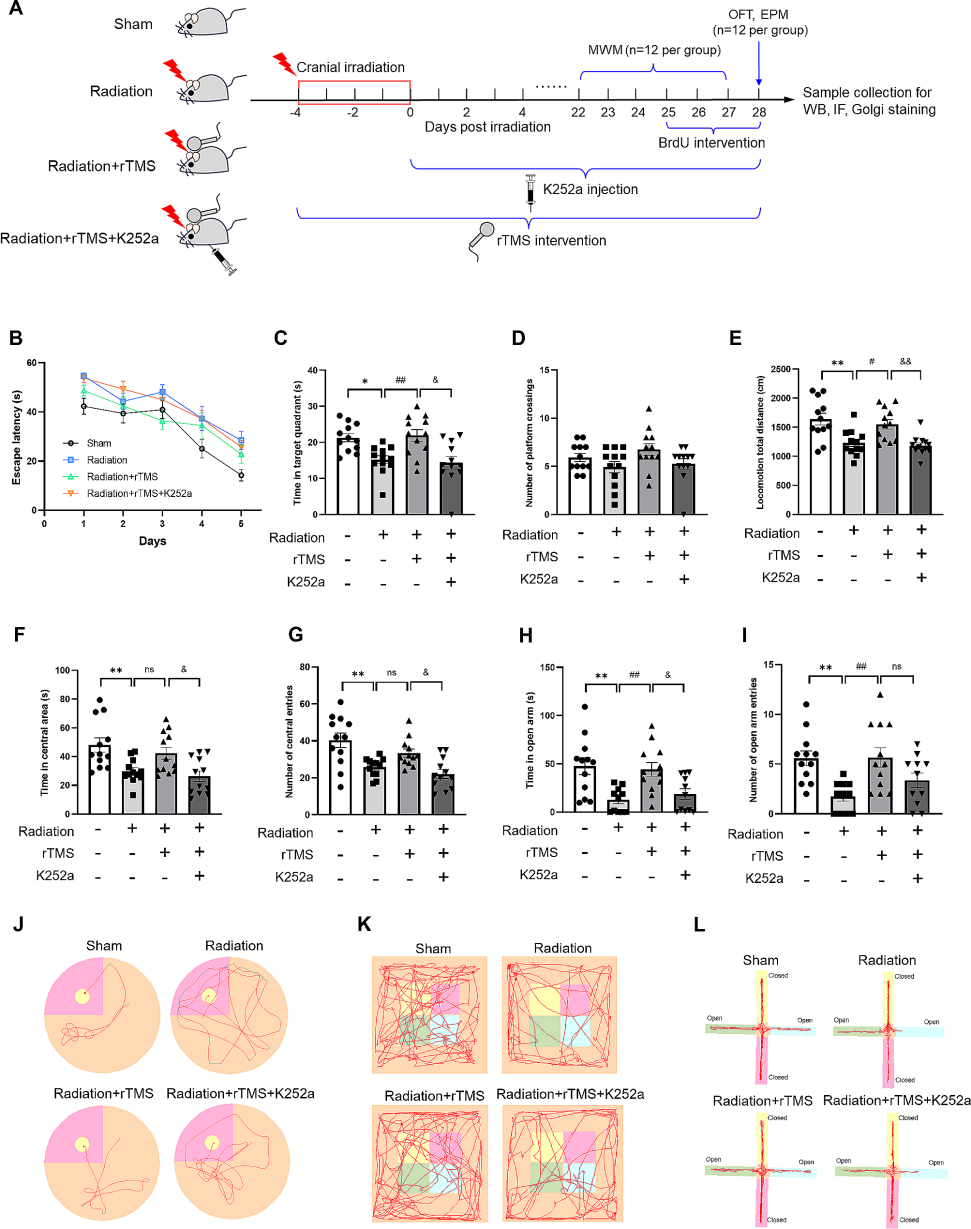
Inhibiting BDNF pathway reversed the improvement of cognitive function and emotionality induced by rTMS in RIBI mice. (**A**) Schematic diagram of the experimental design, including animal grouping, drug administration, intervention protocol, behavioral tests and other experiments. (**B**) The escape latency during navigation test phase. (**C**) The time spent in the target quadrant during probe test phase. (**D**) The number of platform crossings during probe trial. (**E**) The locomotion total distance in open field. (**F**) The total time spent in central area of open field. (**G**) The frequency of central entries in open field. (**H**) The time spent in open arms of elevated plus maze. (**I**) The frequency of open arms entries. (**J**) Representative MWM training trace of mice in each group. (**K**) Representative movement trace of mice in the open field, the four squares in the middle represent central area. (**L**) Representative movement trace of mice in the elevated plus maze, open and closed arms are indicated in the diagrams. Data are presented as mean ± S.E.M, *n* = 11 or 12 for each group. ^*^P < 0.05, ^**^P < 0.01 vs. Sham group; ^#^*P* < 0.05, ^##^*P* < 0.01 vs. Radiation group; ^&^*P* < 0.05, ^&&^*P* < 0.01 vs. rTMS group; ns. no significance

In order to further investigate whether BDNF pathway played an essential role in the protective effects of rTMS on cognition and emotionality in RIBI mice, we blocked BDNF pathway by injecting K252a intraperitoneally, and detected the cognitive function and emotionality alteration (Fig. 4A). The results of MWM showed that after intervention of K252a, the mice exhibited an impaired spatial learning ability, as evidenced by much longer time targeting the platform in training phase compared with rTMS intervention group (Fig. 4B, J). Furthermore, in the space exploration test, compared with rTMS group, the mice in K252a intervention group showed significant time reduction in the target quadrant (Fig. 4C), the number of platform crossings also decreased, while the difference was not statistically significant (Fig. 4D). The results above indicated that inhibiting BDNF pathway by K252a could attenuate the protective effect of rTMS intervention, and exacerbate cognitive deficits and anxiety behavior in RIBI mice.

After inhibiting BDNF pathway, the mice in K252a group showed a significant decline in locomotion distance in open field compared with rTMS intervention group (Fig. 4E). Besides, the results showed that the mice treated with K252a spent less time in central area and decreased the number of central entries (Fig. 4F, G, K). The results of EPM showed that compared with rTMS intervention group, the mice treated with K252a showed a significant decrease in the time of open arms exploration, the number of open arms entries also decreased, while no significant differences were found (Fig. 4H, I, L). These results suggested that after inhibiting BDNF pathway, the improvement of behavioral performance induced by rTMS in RIBI mice was obviously reversed.

### rTMS regulated hippocampal neurogenesis through BDNF pathway

**Fig. 5:**
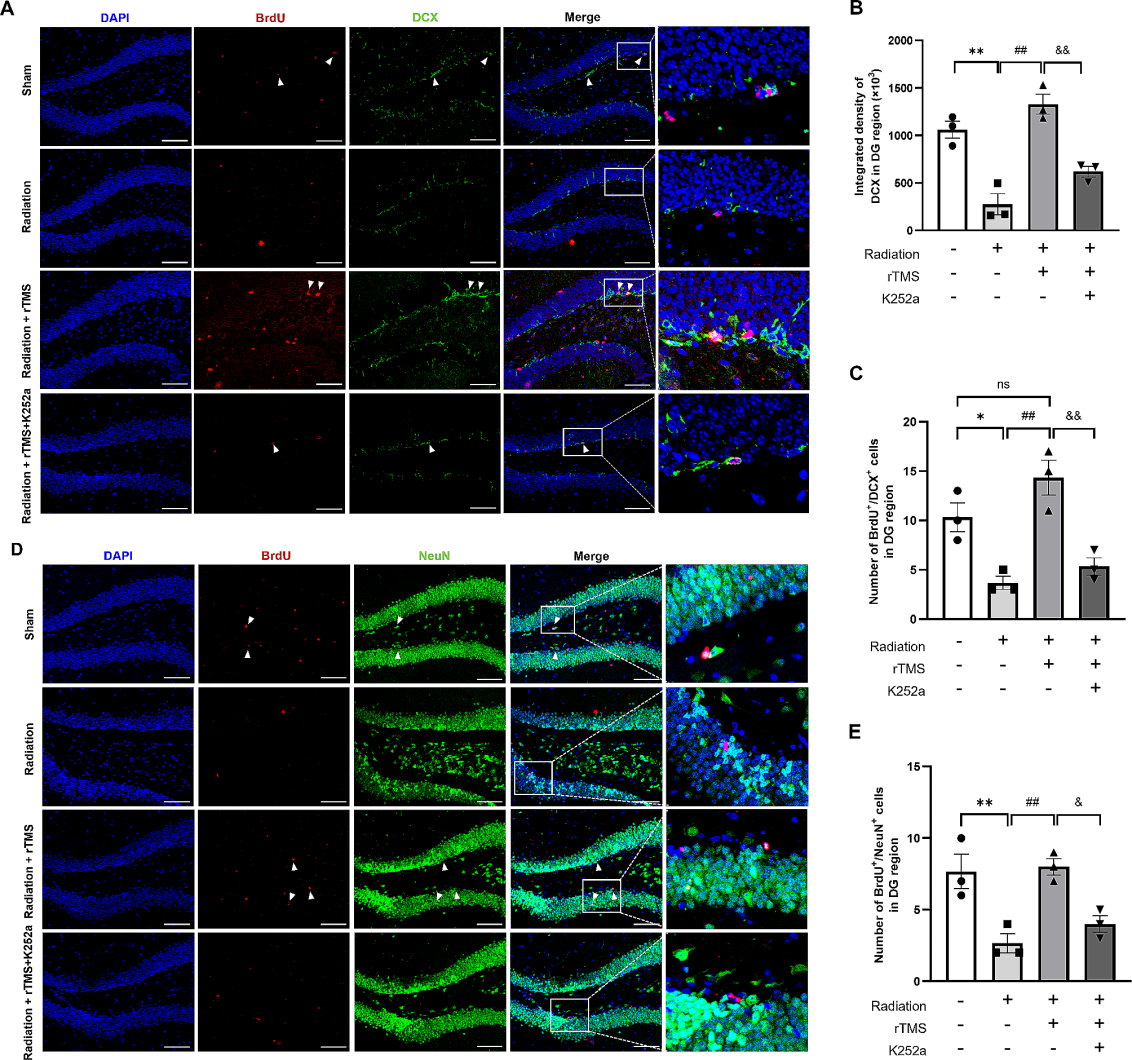
Inhibiting BDNF pathway attenuated the level of hippocampal neurogenesis after rTMS treatment. (**A**) Representative images of immunofluorescence staining of DCX (green)/BrdU (red) in hippocampal sections from Sham and cranial irradiation mice treated with rTMS and K252a. Scale bar = 100 μm. (**B**) Integrated density of DCX in DG region of hippocampus. (**C**) The number of DCX/BrdU positive cells in DG. (**D**) Representative images immunofluorescence staining of NeuN (green)/BrdU (red) in hippocampal sections. Scale bar = 100 μm. (**E**) The number of NeuN/BrdU positive cells in DG of hippocampus. All data are presented as mean ± S.E.M, *n* = 3 for each group. ^*^P < 0.05, ^**^P < 0.01 vs. Sham group; ^##^*P* < 0.01 vs. Radiation group; ^&^*P* < 0.05, ^&&^*P* < 0.01 vs. rTMS group; ns. no significance

Next, we investigated the role of BDNF pathway in hippocampal neurogenesis in RIBI mice. The results of IF staining (Fig. 5A, D) showed that rTMS intervention could obviously enhance the fluorescence density of DCX in DG region of hippocampus compared with that of radiation group (Fig. 5B), and the number of BrdU^+^/DCX^+^ cells in DG significantly increased (Fig. 5C). After blocking BDNF pathway, it was found that the promotion of hippocampal neurogenesis was significantly reversed, which was manifested by the significant decrease in the number of BrdU^+^/DCX^+^ and BrdU^+^/NeuN^+^ cells in DG region (Fig. 5E), as well as the decrease in the density of DCX. These results suggested that blocking BDNF pathway could reverse the promotion of hippocampal neurogenesis induced by rTMS in RIBI mice.

### rTMS mitigated microglial pyroptosis through BDNF pathway

**Fig. 6:**
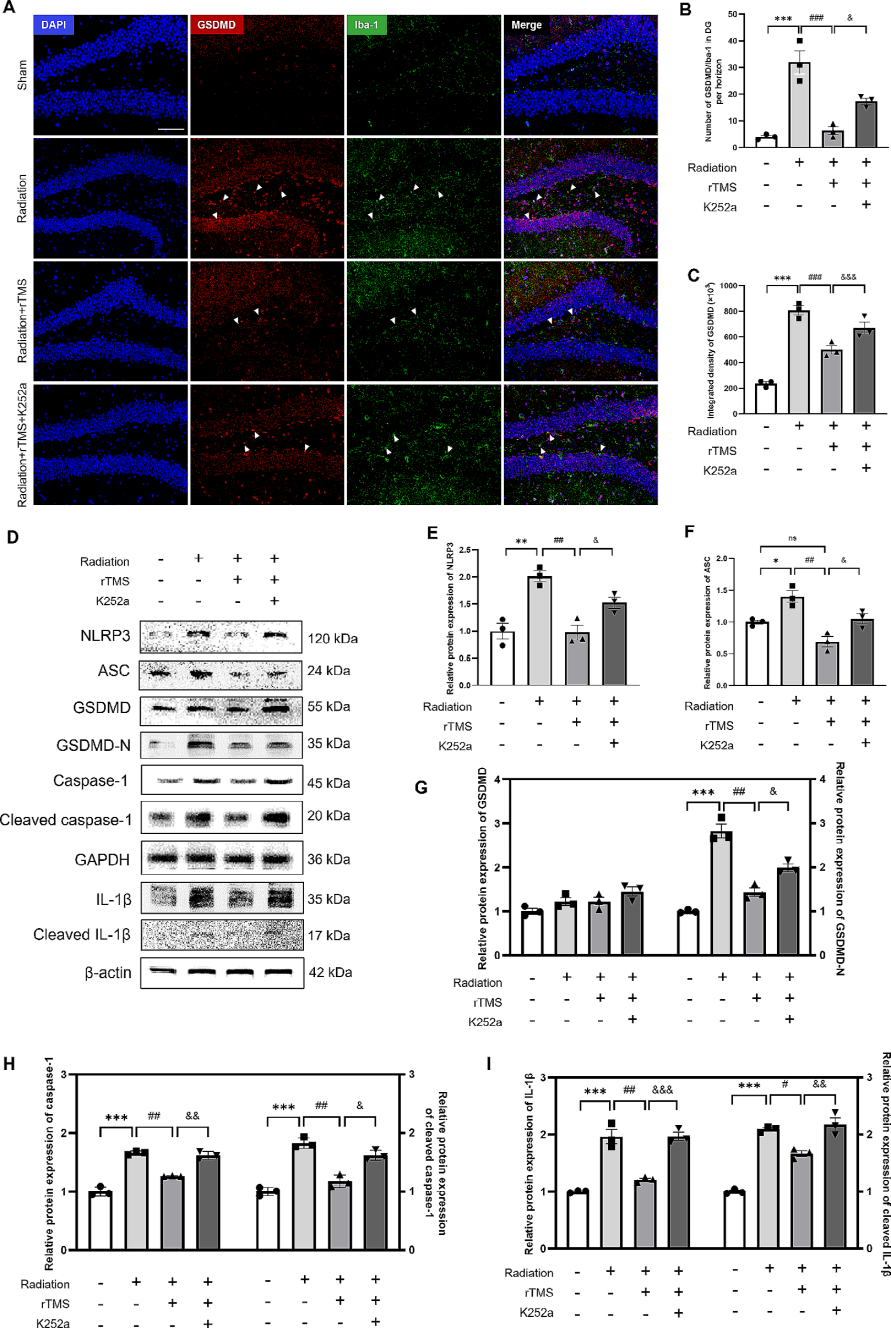
Inhibiting BDNF pathway reversed the anti-pyroptosis effect of rTMS. (**A**) Representative immunofluorescence double staining images of Iba-1 and GSDMD, arrows indicated co-localization of GSDMD with Iba-1. Scale bar = 100 μm. (**B**) The number of GSDMD/Iba-1 positive cells in DG. (**C**) Quantitative analysis of integrated intensity of GSDMD. (**D**) Representative bands of western blotting. (**E-I**) Quantitative analysis of protein expression level by one-way ANOVA followed by Tukey’s multiple comparison test. Data are presented as mean ± S.E.M, *n* = 3 for each group. ^*^P < 0.05, ^**^P < 0.01, ^***^P < 0.001 vs. Sham group; ^#^*P* < 0.05, ^##^*P* < 0.01, ^###^*P* < 0.001 vs. Radiation group; ^&^*P* < 0.05, ^&&^*P* < 0.01, ^&&&^*P* < 0.001 vs. rTMS group; ns. no significance

To address whether BDNF pathway involved in the anti-pyroptosis effect of rTMS, we blocked BDNF pathway and further observed the level of pyroptosis. The results of double-labeled immunofluorescence (Fig. 6A) showed that, compared with rTMS intervention group, the number of GSDMD^+^/Iba-1^+^ cells in DG significantly increased, besides, the integrated intensity of GSDMD significantly enhanced after inhibiting BDNF pathway (Fig. 6B, C). Consistently, we also detected the protein expression levels of NLRP3 inflammasome, GSDMD, caspase-1 and IL-1β in hippocampus of mice (Fig. 6D). We found significant higher immunoreactivity levels of NLRP3, ASC, GSDMD-N, caspase-1 and IL-1β in K252a group than that of rTMS intervention group (Fig. 6E-I). Therefore, the above results suggested that rTMS may modulate inflammatory response through BDNF pathway, as expected, inhibition of BDNF pathway obviously reduced the protective and anti-pyroptosis effects of rTMS.

### rTMS enhanced synaptic plasticity through BDNF pathway

**Fig. 7:**
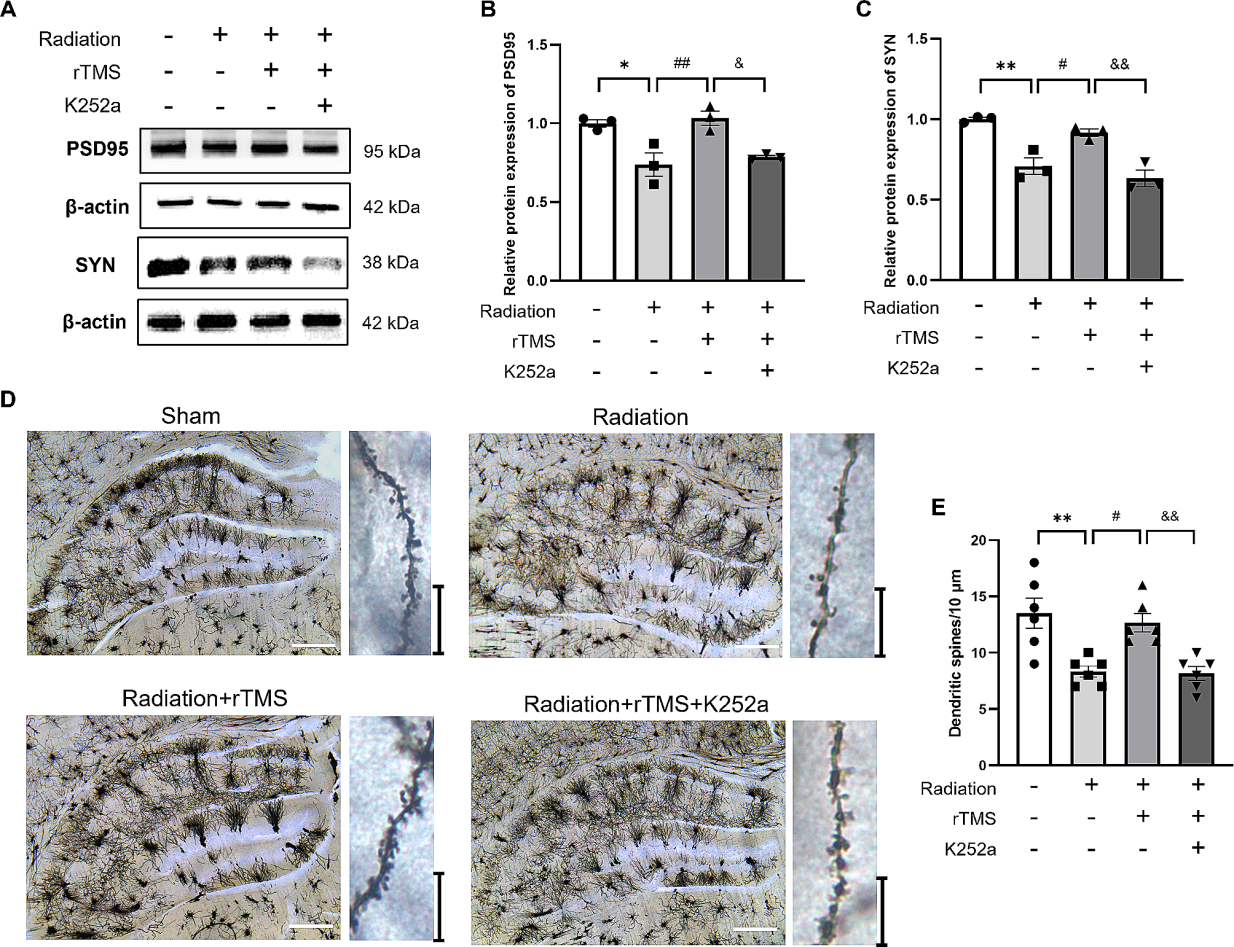
Inhibiting BDNF pathway reversed the enhancement of synaptic plasticity after rTMS treatment. (**A-C**) The protein levels of PSD95 and Synaptophysin, *n* = 3 for each group. (**D**) Representative images of Golgi staining and tertiary dendritic profile of granule cells in DG region of hippocampus. Scale bar = 200 μm for images of hippocampus; and scale bar = 10 μm for tertiary dendritic images. (**E**) The spines density of tertiary dendritic per horizons. *n* = 2 for each group and 6 horizons were randomly chosen for analysis. All data are presented as mean ± SEM, ^*^P < 0.05, ^**^P < 0.01 vs. Sham group; ^#^*P* < 0.05, ^##^*P* < 0.01 vs. Radiation group; ^&^*P* < 0.05, ^&&^*P* < 0.01 vs. rTMS group

Synaptic plasticity plays a pivotal role in regulating cognitive function. We previously proved that RIBI mice showed an impairment of ultrastructure of synapses and decreased number of neuronal synapses. In order to better reveal the alteration of synaptic plasticity after blocking BDNF pathway, western blotting and Golgi staining were performed to detect neuronal synaptic plasticity. The results of western blotting showed that the levels of PSD95 and synaptophysin (SYN) were significantly downregulated after BDNF pathway was inhibited, compared with rTMS group (Fig. 7A-C), which indicated that K252a could reverse the effect of rTMS in the increase of synapse-related proteins expression in RIBI mice. Then we further performed Golgi staining to assess the structural plasticity. The representative image of tertiary dendritic profile of granule cells in DG region of hippocampus was shown in Fig. 7D. The results of quantitative analysis showed that the dendritic spines of radiation group were significantly decreased compared with that of in Sham group (Fig. 7E), while it obviously increased in rTMS intervention group. After BDNF pathway was blocked, the dendritic density was evidently declined compared with rTMS intervention group. The results suggested that K252a could reverse the enhancement of synaptic plasticity induced by rTMS, therefore, BDNF pathway exerted an essential role in regulating synaptic plasticity in hippocampus.

## Discussion

RIBI is a common complication of cranial radiotherapy and it gives rise to serious adverse effects on patients. Numerous studies believed that multiple factors contributed to RIBI, including hippocampal neurogenesis impairment [26], increasing neuroinflammatory response [27, 28] and oxidative stress, free radical damage theory and BBB disruption [29] et al. BDNF is closely related to the level of neurogenesis and synaptic plasticity, investigating the potential mechanisms of BDNF is of great importance. We previously found that the BDNF expression level significantly increased after rTMS intervention in RIBI mice [15], while the specific mechanism of rTMS on RIBI has not been well clarified. Hence, in this study, we provided evidence that rTMS improved cognitive function in RIBI mice model through regulating hippocampal neurogenesis and neuroinflammation, and BDNF signaling pathway was participating in it.

We had proved that high-frequency rTMS significantly reduced cognitive dysfunction in RIBI mice, while its mechanism was still unclear. BDNF signaling pathway, as an essential functional pathway in the brain, plays a crucial role in the development of neurons, and the formation and consolidation of learning and memory [30, 31]. In order to figure out whether BDNF pathway took part in regulating cognition in RIBI mice, we inhibited BDNF pathway by injecting K252a, we found that the spatial learning and memory ability of mice significantly decreased through MWM test. Furthermore, the results of OFT and EPM indicated that the mice exhibited anxiety-like behavior after BDNF pathway was inhibited, which suggested that BDNF pathway may be involved in regulating learning and memory ability and emotionality in mice. Similarly, it was reported that high-frequency (5 Hz) rTMS intervention could significantly alleviate the impairment of spatial learning and memory ability in the offspring of prenatal stressed rats, while after administration of K252a could significantly antagonize the effect of rTMS [32], which was consistent with the present study. Besides, another study [33] also reported that blocking BDNF pathway by consecutively injection of K252a could significantly inhibit the antidepressant effect of L701324 (an NMDA receptor) in chronic unpredictable mild stress (CUMS) mice, and decrease BDNF and CREB protein expression levels. Based on previous researches and our findings, we believe that K252a specifically blocks BDNF pathway, so as to antagonize the therapeutic effect of rTMS on cognition and emotionality. Therefore, these results suggested that BDNF pathway is indispensable for rTMS in alleviating cognitive dysfunction and anxiety behavior in RIBI mice.

Hippocampal neurogenesis refers to the process in which NSCs proliferate and migrate to genesis center, then differentiate into mature neurons and glial cells, it mainly occurs in the DG region of hippocampus and SVZ [34], DCX and NeuN are widely believed to be important markers for neurogenesis. Previous studies had proved that rTMS could significantly improve the level of neurogenesis, which was related to the upregulation of BDNF [9, 35]. Besides, some studies found that high frequency (10–20 Hz) rTMS could promote neurogenesis in rats with ischemic stroke, and the activation of BDNF signaling pathway may be involved in the mechanism [13, 36]. Consistent with these studies, our results showed that rTMS could significantly increase the number of ki67 and DCX in hippocampus of RIBI mice, which indicated that rTMS promoted hippocampal neurogenesis after cranial irradiation. Based on previous studies and our findings, we speculated that rTMS obviously promoted hippocampal neurogenesis in RIBI mice, and BDNF signaling pathway might play an essential role in it. To further verify the specific role of BDNF pathway in neurogenesis, we then inhibited BDNF pathway by intraperitoneal injection of K252a, and found that the level of hippocampal neurogenesis was reversed by K252a. The current studies also showed similar results [37–39]. In addition, Pei et al. revealed that electroacupuncture stimulation could promote hippocampal neurogenesis through activating BDNF-TrkB pathway, so as to alleviate spatial memory damage induced by sleep deprivation in rat [40]. Moreover, another research conducted by Chen et al. [41] found that Chaihu Shugan San (CSS) showed a therapeutic role in depressive-like rats exposed to CUMS and increased hippocampal neurogenesis, however, K252a fully attenuated the role of CSS, which verified that BDNF pathway was required for the neuronal proliferation, and revealed that promoting neurogenesis might be a potential strategy for treating depression in adult animals. Thus, based on what we have found in this study, we believed that rTMS could promote hippocampal neurogenesis in RIBI mice which was associated with BDNF signaling pathway.

**Fig. 8:**
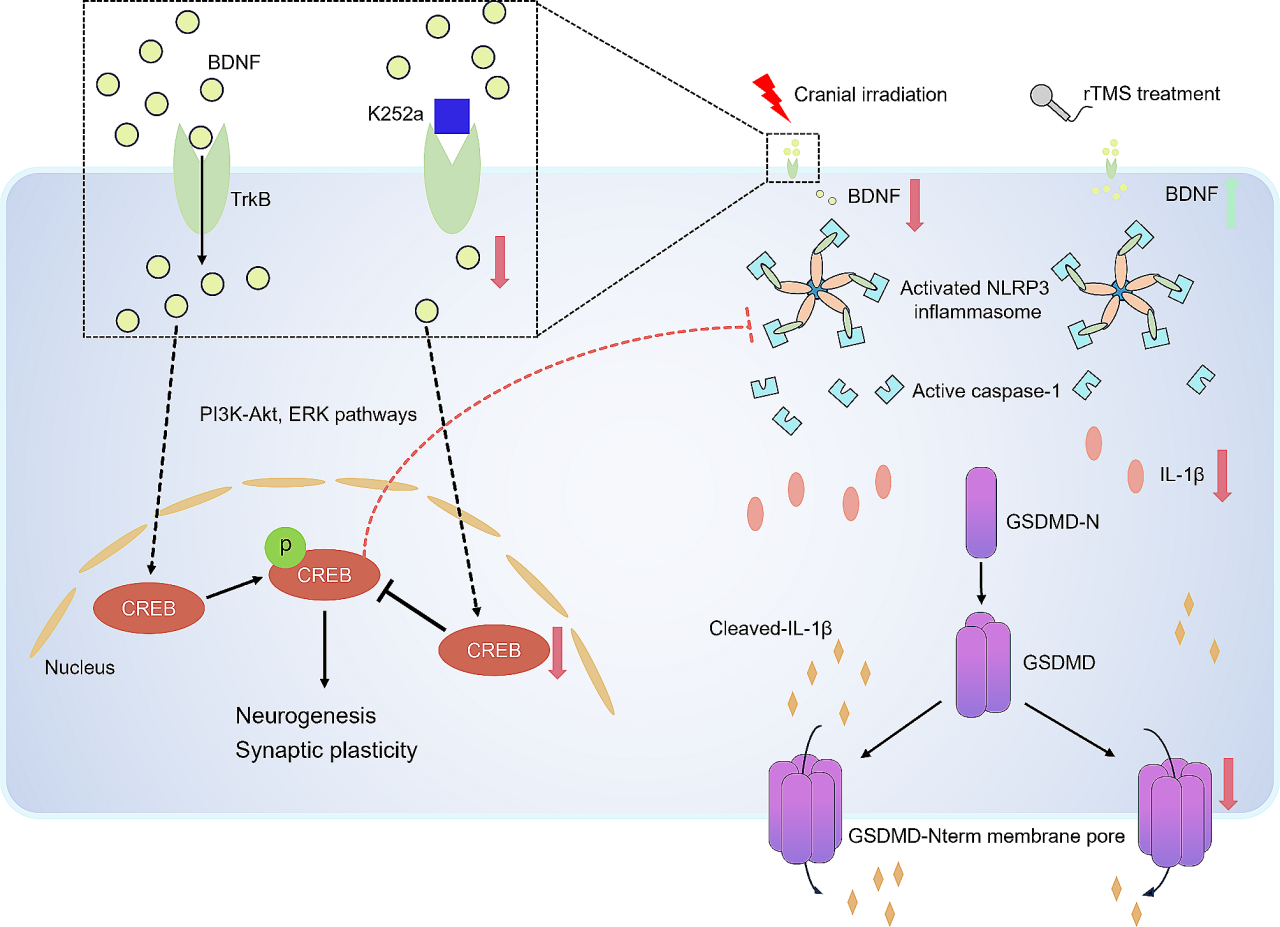
The hypothesized schematic diagram of possible relation between BDNF and NLRP3-mediated pyroptosis in RIBI

Previous studies have shown that the activation of NLRP3 inflammasome and the release of inflammatory factors are closely related to the cell pyroptosis [42, 43], microglia are important immune cells in CNS, which is considered to be the main cell type in pyroptosis [44]. The overactivation of microglia leads to an increase in neuroinflammation, resulting in dysfunction in CNS [45]. To explore the effect of rTMS on neuroinflammation, we observed the status of microglia in hippocampus, and found that rTMS intervention could inhibit microglial activation induced by cranial irradiation. In addition, the number of GSDMD^+^/Iba-1^+^ cells in DG region of hippocampus were found to increase significantly after cranial irradiation, while there was no obvious co-localization of GSDMD/GFAP and GSDMD/NeuN, which indicated that cell pyroptosis induced by cranial irradiation mainly occurred in microglia, instead of astrocyte and neuron. Although we have found microglial pyroptosis may be involved in the neuroinflammation, the potential regulating mechanism have not been well clarified. It was reported that NLRP3 inflammasome consisted of three protein subunits, NLRP3, ASC and caspase-1 [20], the activity of NLRP3 inflammasome has been considered as one of the important factors in a variety of CNS diseases, such as Alzheimer’s disease and stroke [46, 47]. Moreover, the activation of microglia-mediated NLRP3 inflammasome and cytokine IL-1β could exacerbate the level of neuroinflammation [48]. In the present study, we found that cranial irradiation could activate NLRP3 inflammasome and upregulate the expression levels of GSDMD as well as proinflammatory cytokine IL-1β in hippocampus of mice, rTMS could suppress the upregulation of these proteins’ expression. Similarly, some scholars revealed that exhaust particulate activated hippocampal microglial cells, which induced NLRP3 inflammasome activation, consequently leading to learning and memory impairment [49]. In addition, Zhou et al. [50] reported that NLRP3/caspase-1/GSDMD axis was involved in sevoflurane induced microglial pyroptosis. A study conducted by Fan et al. found that inhibiting NLRP3 could significantly decrease the level of NLRP3 inflammasome and IL-1β in hippocampus of mice and significantly alleviated cognitive dysfunction [51]. Therefore, NLRP3 inflammasome played a critical role in neuroinflammation, and we speculated that cranial irradiation caused microglial pyroptosis induced by NLRP3 inflammasome. Next, in order to further explore whether BDNF pathway plays an important role in neuroinflammation after rTMS intervention, we inhibited BDNF pathway and observed the level of cell pyroptosis. We found that after blocking BDNF pathway, the level of cell pyroptosis in hippocampus increased obviously, as well as the increase of NLRP3 inflammasome and IL-1β. Our findings suggested that blocking BDNF pathway suppressed the therapeutic effect of rTMS, which indicated that rTMS ameliorated neuroinflammation through BDNF pathway in mice. Based on the findings in recent studies, we believed that rTMS alleviated microglial pyroptosis mediated by NLRP3 inflammasome after cranial irradiation, and BDNF pathway may be involved in it. Although BDNF is closely associated with neuronal development and synaptic plasticity, the molecular mechanism of BDNF in neuroinflammation, NLRP3 activation specifically, have not been well clarified. However, according to Alexandria et al’s study [52], BDNF signaling activated PI3K-Akt signaling pathway as well as MEK pathway, the former then activated CREB and its phosphorylation. Similar explanation was shown in Li’s study [12], they revealed that the downstream targets of BDNF-TrkB signaling including p-ERK, p-CREB, and p-Akt, which were pivotal to neuroinflammation, neuronal survival. In Zhen’s study in 2023 [53], they believed ERK/CREB pathway was essential for oxidative stress and neuroinflammatory processes. In brief, based on previous studies, we speculate BDNF might mediate NLRP3 activation through activating ERK-CREB or PI3K-AKT-CREB signaling pathway (Fig. 8).

Synaptic plasticity is the basis of learning and memory formation, which mainly includes structural and functional ability. SYN and PSD95 are typical markers of synaptic proteins, which are associated with aging and radiation [54]. We previously found that rTMS could upregulate the expression of PSD95 and SYN in RIBI mice, as well as significantly ameliorate the ultrastructural damage of synapses [15]. In order to explore the role of BDNF in synaptic plasticity, we blocked the BDNF pathway by K252a and observed the level of synapse-related proteins. It was found that the protein expression levels of PSD95 and SYN were significantly downregulated, which implied that BDNF pathway might be an important factor for synaptic structure and function. Similar research had shown that low-frequency (1 Hz) rTMS improved cognitive function in patients with stroke, and regulated synaptic plasticity through BDNF signaling pathway [55]. In addition, rTMS could activate BDNF pathway and upregulate the expression levels of SYN, PSD95 and GAP43 to enhance the activity of synapses [56]. Dendritic spines are the main parts of synaptic formation and play a pivotal role in information transmission between neurons, the density of which is closely related to the development of learning and memory [57]. It has been reported that rTMS remarkedly alleviated learning and memory ability impairment caused by hindlimb unloading mice, which was associated with increasing spine density in hippocampus, and BDNF-TrkB pathway might be responsible for it [58]. Similar to these findings, to further investigate the possible role of BDNF pathway in structural plasticity, we performed Golgi staining to observe the morphology of dendritic spines in hippocampus, the results indicated that after blocking BDNF pathway, the density of dendritic spines obviously decreased. Our finding indicated that BDNF pathway might play an important role in synaptic regulation, K252a could reverse the protective effect of rTMS in synaptic plasticity. Therefore, we concluded that rTMS may enhance hippocampal synaptic plasticity via mediating BDNF pathway.

This study has some limitations. Firstly, we only included male mice in the study, the results of female mice had not been demonstrated, the gender factor might be a potential experimental bias in the present results. Secondly, the optimal parameter of rTMS, such as frequency, intensity and the number of pulses, for the treatment of RIBI was not studied. Future studies are warranted to explore the optimal parameters of rTMS, so as to achieve better therapeutic effect. Finally, our results only investigated synaptic plasticity in structure, but did not perform functional plasticity, which needed to be explored in further studies.

## Conclusion

Taken together, our study suggests that rTMS ameliorated cognitive deficts in RIBI mice by improving neurological deficits and mitigating neuroinflammation through regulating BDNF signaling pathway. The findings may provide novel inspiration for clinical treatment of brain injury induced by cranial radiotherapy.

### Electronic supplementary material

Below is the link to the electronic supplementary material.

Supplementary Material 1

## Data Availability

All data generated or analysed during this study are included in this published article.

## Abbreviations

ANOVA: Analysis of variance
BBB: Blood brain barrier
BDNF: Brain-derived neurotrophic factor
BrdU: 5-bromo-2’-deoxyuridine
CNS: Central nervous system
CUMS: Chronic unpredictable mild stress
EPM: Elevated plus maze test
GSDMD: Gasdermin D
IF: Immunofluorescence
IHC: Immunohistochemistry
LTP: Long-term potentiation
MWM: Morris water maze
NSCs: Neural stem cells
OFT: Open field test
PSD95: Postsynaptic density 95
PTSD: Post-traumatic stress disorder
RIBI: Radiation-induced brain injury
ROS: Reactive oxygen species
rTMS: Repetitive transcranial magnetic stimulation
SYN: Synaptophysin
