# Supplementary material for: Repetitive transcranial magnetic stimulation ameliorates cognitive deficits in mice with radiation-induced brain injury by attenuating microglial pyroptosis and promoting neurogenesis via BDNF pathway
Source: Cell Commun Signal. 2024 Apr 3;22:216. doi: 10.1186/s12964-024-01591-0 (PMC10988892; doi:10.1186/s12964-024-01591-0)
Supplement: Supplementary file 1 — Supplementary Material 1 [file 12964_2024_1591_MOESM1_ESM.docx]

**Additional file**

**Table S1.** Details of antibodies used in WB and IF in the study.

| **Antibody** | **Brand** | **Catalogue number** | **Host species** | **Application** |
| --- | --- | --- | --- | --- |
| BDNF | Cell Signaling Technology, USA | 47808 | rabbit | WB 1:1000 |
| TrkB | Cell Signaling Technology, USA | 4603 | rabbit | WB 1:1000 |
| p-TrkB | Abclonal, USA | AP0423 | rabbit | WB 1:1000 |
| CREB | Cell Signaling Technology, USA | 9197 | rabbit | WB 1:2000 |
| p-CREB | Cell Signaling Technology, USA | 9198 | rabbit | WB 1:1000 |
| GSDMD | Affinity, USA | AF4012 | rabbit | WB 1:1000 |
| NLRP3 | Affinity, USA | DF15549 | rabbit | WB 1:1000 |
| Caspase-1 | Abcam, UK | ab179515 | rabbit | WB 1:2000 |
| ASC | Cell Signaling Technology, USA | 67824 | rabbit | WB 1:2000 |
| IL-1β | Proteintech, USA | 26048-1-AP | rabbit | WB 1:2000 |
| Synaptophysin | Sigma, USA | S5768 | mouse | WB 1:2500 |
| PSD95 | Cell Signaling Technology, USA | 3450 | rabbit | WB 1:2000 |
| β-actin | Cell Signaling Technology, USA | 4970 | rabbit | WB 1:3000 |
| DCX | Abcam, USA | ab207175 | rabbit | IF 1:300 |
| Ki67 | Abcam, USA | ab16667 | rabbit | IF 1:250 |
| GSDMD | Affinity, USA | AF4012 | rabbit | IF 1:200 |
| Iba-1 | GeneTex, USA | GTX632426 | mouse | IF 1:150 |
| NeuN | Abclonal, USA | A19086 | rabbit | IF 1:200 |
| BrdU | Abcam, USA | ab6326 | rat | IF 1:250 |


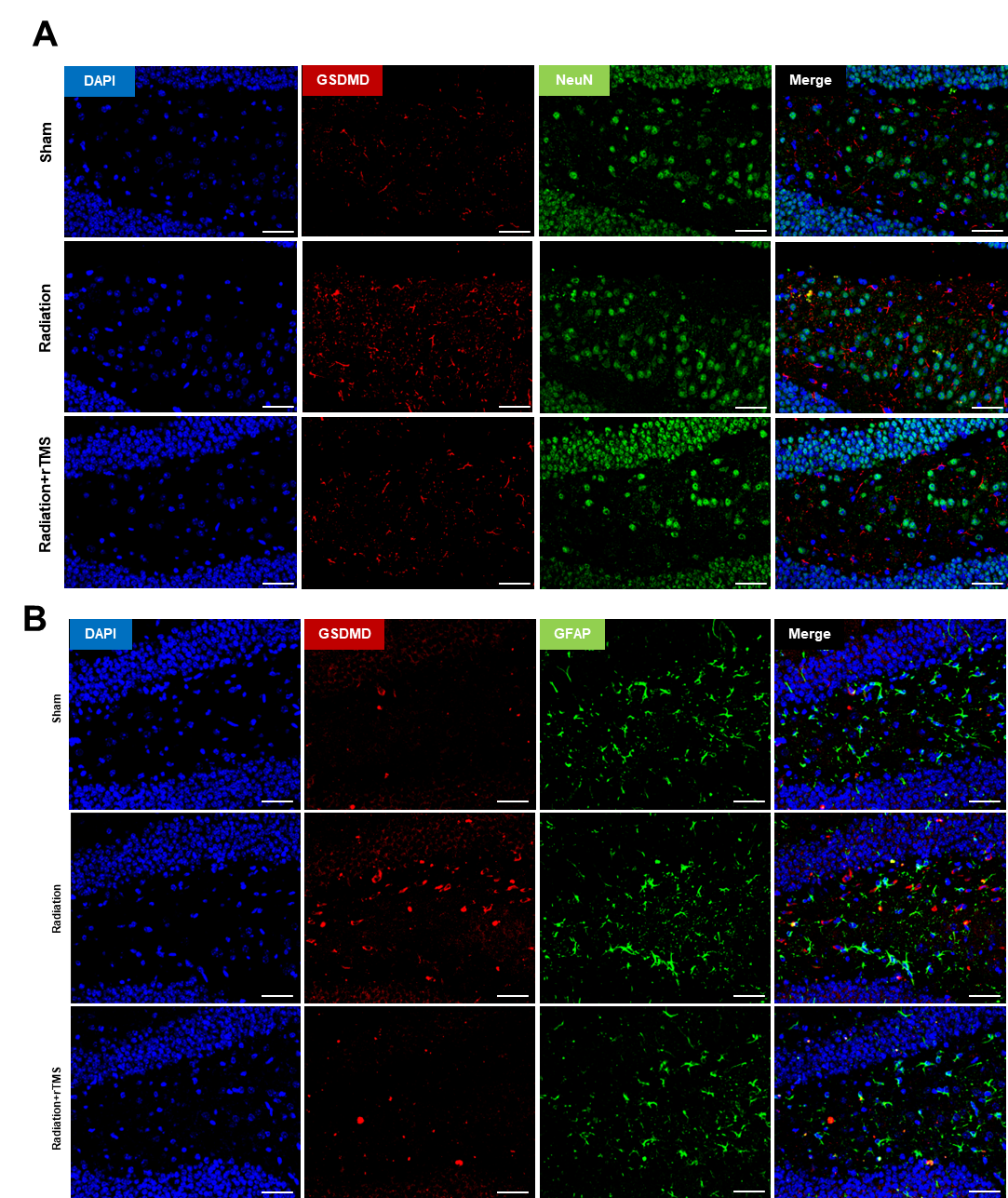


**Figure S1.** The immunofluorescence staining of GSDMD/NeuN **(A)** and GSDMD/GFAP **(B)** in hippocampus of mice. The results above indicated that there were no obvious double-labeled positive cells between GSDMD and NeuN or GFAP. Scale bar = 50 μm.
